# In Situ Electrochemically Generating High-Valent Iron Species Activated by Nitrogen-Doped Biochar for Efficient Degradation of Antibiotics

**DOI:** 10.3390/antibiotics15030254

**Published:** 2026-03-01

**Authors:** Yuhang Lin, Anting Ding, Zhikang Deng, Ya-Nan Zhang, Chenyu Zeng, Fuyu Xie, Yumu Luo, Minle Li, Junwei Ma, Zulin Zhang

**Affiliations:** 1 Xianghu Laboratory, Hangzhou 311231, China; 2120230584@glut.edu.cn (Y.L.);; 2 School of Resources and Environmental Engineering, Wuhan University of Technology, Wuhan 430070, China; 3 Guangxi Key Laboratory of Environmental Pollution Control Theory and Technology, Guilin University of Technology, Guilin 541006, China; 4 University Engineering Research Center of Watershed Protection and Green Development, Guilin University of Technology, Guilin 541006, China; 5 Institute of Environment, Resource, Soil and Fertilizers, Zhejiang Academy of Agricultural Sciences, Hangzhou 310021, China; 6 The James Hutton Institute, Craigiebuckler, Aberdeen AB15 8QH, UK

**Keywords:** electrogenerated high-valent iron, SMX, N-doped biochar, electron transfer, ROS

## Abstract

**Background:** Traditional methods exhibit an extremely low removal efficiency for antibiotics in water, making an efficient and energy-saving approach urgently needed. **Methods and Results:** In this study, a novel catalytic approach based on the in situ generation of high-valent iron (Fe(IV)/Fe(V)) has been developed by adding biochar instead of modifying the electrode materials (in previous studies) for the efficient removal of sulfamethoxazole (SMX) from water. Fe(IV)/Fe(V) was produced by the anodic oxidation of low concentrations of Fe(III) and subsequently activated by nitrogen-doped corn stalk biochar (NBC). The results showed that the degradation efficiency increased from 50.83% to 90.67% within 60 min after the addition of nitrogen-modified biochar. The abundant defect structures, graphitic N and oxygen-containing functional groups in NBC endowed the catalyst with excellent activation capability. Quenching experiments and methyl phenyl sulfoxide (PMSO) probe experiments revealed that singlet oxygen (^1^O_2_) and Fe(IV)/Fe(V) were the main contributors to SMX degradation. Degradation pathways were inferred based on transformation products (TPs) and density functional theory (DFT) calculations. Ecotoxicity prediction using the ECOSAR program indicated that the TPs formed in the E/Fe(III)/NBC system exhibited markedly lower toxicity to aquatic organisms than the parent SMX. Furthermore, the E/Fe(III)/NBC system maintained a high degradation efficiency for SMX in real aquatic environments. Additionally, the E/Fe(III)/NBC system showed high removal rates for other sulfonamides such as sulfadiazine (SDZ), sulfamethoxypyridazine (SMP), sulfathiazole (STZ) and sulfadoxine (SDX). **Conclusions:** Overall, the E/Fe(III)/NBC system was demonstrated to be a highly efficient and sustainable technology for removing various antibiotics from water.

## 1. Introduction

The natural antibiotic Penicillin was first discovered in 1929 [1]. Due to its outstanding antibacterial properties, humans have continuously deepened research into extracting natural antibiotics and modifying them at the molecular level, deploying hundreds of antibiotics in medical clinical settings, aquaculture, and animal husbandry. In recent years, global antibiotic consumption has increased by 65% [2]. China, as a populous nation, is also a major producer and consumer of antibiotics. By 2023, annual antibiotic usage exceeded 180,000 tons (China Report Center). However, limited utilization rates in both human and animal applications resulted in 50–90% of antibiotics entering the environment as parent compounds or through excretion [3], which caused antibiotic pollution in water bodies. Among these antibiotics, sulfamethoxazole (SMX) was one of the most widely used typical antibiotics, which was employed for urinary tract infections and avian influenza [4]. However, its overuse and even misuse have led to the frequent detection of high-concentration residues in various aquatic environments.

SMX inhibits bacterial metabolism by competitively binding to dihydrofolate synthase, thereby inhibiting the conversion of para-aminobenzoic acid esters into dihydrofolate, which was a precursor to tetrahydrofolate and essential for nucleic acid synthesis [5]. Conventional treatment methods, such as coagulation, activated sludge processes, and membrane separation, generally fail to achieve the efficient or complete removal of SMX [6]. SMX, when presented in water at high concentrations over extended periods, could alter microbial communities and aquatic ecosystems, induce bacterial resistance, and spread antibiotic resistance genes [7]. Therefore, developing an efficient and sustainable degradation strategy for SMX is of significant environmental importance. Traditional treatment techniques such as adsorption and biofilm-based processes often suffer from limited degradation efficiency, complex operation, and high maintenance costs. Comparatively, advanced oxidation processes (AOPs) provide high mineralization efficiency, primarily due to disrupting the structure of target pollutants in a short timeframe, simultaneously converting them into smaller molecular species or less toxic substances [8]. In AOPs, exogenous oxidants played a crucial role. Common exogenous oxidizing agents include peroxymonosulfate (PMS), peroxydisulfate (PDS), peracetic acid (PAA) [9], permanganate (KMnO_4_) and ferrate (Fe(VI)) [10]. In particular, activated ferrate (Fe(VI)) technology, which converted Fe(VI) to Fe(IV)/Fe(V), had attracted extensive research, because of the higher reactivity toward pollutants than other oxidants. Previous studies have generally relied on the external addition of Fe(VI) at relatively high dosages, followed by activation through electrochemical processes or carbonaceous materials to enhance Fe(IV)/Fe(V) generation. However, the exogenous addition of ferrate (Fe(VI)) would increase the operational costs and may introduce secondary pollution risks. Recent studies have demonstrated that Fe(IV)/Fe(V) could be in situ-generated at the anode through the oxidation of Fe(III) in electrochemical processes, which provided a feasible pathway to avoid reliance on externally supplied oxidants. Fe(III) is naturally abundant in many surface waters at concentrations ranging from 12.5 to 25 μM, which provided an available precursor for Fe(IV)/Fe(V). Similarly, studies have employed the electrochemical in situ generation of persulfate ions [11], while Fe(IV)/Fe(V) exhibit higher selectivity toward target pollutants and superior stability compared to them.


(1) $$\mathrm{Fe}(\mathrm{III})\overset{{1e}^{-}\mathrm{transfer}\;\left(\mathrm{anode}\right)}{\to }\mathrm{Fe}(\mathrm{IV})$$

(2) $$\mathrm{Fe}(\mathrm{III})\overset{{2e}^{-}\mathrm{transfer}\;\left(\mathrm{anode}\right)}{\to }\mathrm{Fe}(V)$$

(3) $$\mathrm{Fe}(\mathrm{III})+\cdot \mathrm{OH}\to \mathrm{Fe}(\mathrm{IV})+{OH}^{-}$$

(4) $$\mathrm{Fe}(\mathrm{IV})+\cdot \mathrm{OH}\to \mathrm{Fe}(V)+{OH}^{-}$$

(5) $$\mathrm{Fe}(\mathrm{IV})\overset{{2e}^{-}\mathrm{transfer}\;\left(\mathrm{anode}\right)}{\to }\mathrm{Fe}(\mathrm{VI})$$

(6) $$\mathrm{Fe}(V)\overset{{1e}^{-}\mathrm{transfer}\;\left(\mathrm{anode}\right)}{\to }\mathrm{Fe}(\mathrm{VI})$$

(7) $$\mathrm{Fe}(V)+\cdot \mathrm{OH}\to \mathrm{Fe}(\mathrm{VI})+{OH}^{-}$$

Generally, as shown in Equations (1)–(7), Fe(III) was oxidized by direct electron transfer processes involving 1e^−^ or 2e^−^ or by ·OH in situ-generated in the electrochemical oxidation system. Fe(IV)/Fe(V) was formed as an intermediate, subsequently yielding Fe(VI). Previous studies [12] mainly focused on enhancing approaches for the efficient generation of Fe(IV)/Fe(V) by improving the electrode materials, while the high manufacturing cost of electrodes makes them impractical for real-world applications. Therefore, enhancing the generation rate of Fe(IV)/Fe(V) through the addition of biochar to achieve the sustainable and efficient removal of target pollutants was innovatively proposed in this study.

Biochar has been widely recognized as a promising activator in advanced oxidation processes due to its wide availability, low cost, and diverse surface functional groups. Corn stalks, as agricultural waste, represent a highly promising feedstock for biochar production. China’s annual output of corn stalks reaches approximately 310 million tons, accounting for nearly 40% of the nation’s total agricultural solid waste. Traditional disposal methods are incineration, which not only pose a high risk of fire but also cause severe pollutants to the atmospheric environment. Eco-friendly disposal technology of corn stalks is to pyrolyze them under anoxic or limited-oxygen conditions. In general, low activation rates limited the practical application of pristine biochar. Thus, the functionality of biochar had been enhanced through various techniques [13] (e.g., acid–base treatment, doping with metals or non-metals, and ball milling) to improve its activation performance. Compared to metal doping, non-metal modification avoids the risk of metal leaching and secondary contamination [14]. Among non-metallic (N, S, P) modifications, nitrogen doping is particularly effective because it modulates the electronic structure of carbon, generates abundant defect sites, and substantially improves both radical and non-radical oxidation pathways [15,16]. Specifically, when nitrogen atoms bearing localized unpaired electrons were loaded onto carbon, they disrupted the original electron distribution of carbon and generated more defects and active sites; on the other hand, they introduced graphitic nitrogen, pyridine nitrogen, and pyrrole nitrogen. Graphite nitrogen, as the primary active site mediating electron transfer, played a crucial role in non-radical oxidation processes [17], facilitating electron transfer and promoting the oxidation of low-valent iron species to high-valent Fe(IV)/Fe(V). Furthermore, the electrochemical conditions can regenerate oxygen-containing functional groups during AOP reactions, thereby ensuring the long-term catalytic stability of biochar (Equations (8)–(11)). Hence, this study proposed that utilizing nitrogen-doped biochar enhanced the Fe(IV)/Fe(V) formation rate and enabled the continuous oxidation of Fe(III) to Fe(IV)/Fe(V), to provide a sustainable and effective approach for the efficient in situ generation of Fe(IV)/Fe(V) to degrade antibiotics.

(8) $$BC-H-e^{-}\to \mathrm{BC}-O\cdot$$

(9) $$\mathrm{BC}-O\cdot +e^{-}\to \mathrm{BC}=O$$

(10) $$\mathrm{BC}-O\cdot +H^{+}+e^{-}\to \mathrm{BC}-\mathrm{OH}$$

(11) $$\mathrm{BC}-\mathrm{COO}\cdot +H^{+}+e^{-}\to \mathrm{BC}-\mathrm{COOH}$$

The aims of this study were to (1) build up the E/Fe(III)/NBC system and investigate its degradation performance of SMX from water, (2) elucidate the degradation mechanism of SMX in the E/Fe(III)/NBC system through characterization analysis, quenching studies, electron paramagnetic resonance (EPR), quantitative assessment of Fe(IV)/Fe(V), and environmental factors (e.g., anion type/concentration, humic acid (HA) concentration, and voltage), (3) investigate the reusability of NBC, (4) evaluate the broad-spectrum degradation of sulfonamides in the E/Fe(III)/NBC system and its degradation performance in an actual aquatic environment, (5) propose potential degradation pathways of SMX based on the identification of TPs and DFT calculations, and (6) compare the biotoxicity of SMX with its TPs.

## 2. Results and Discussion

### 2.1. Characterization of Biochar

**Figure 1:**
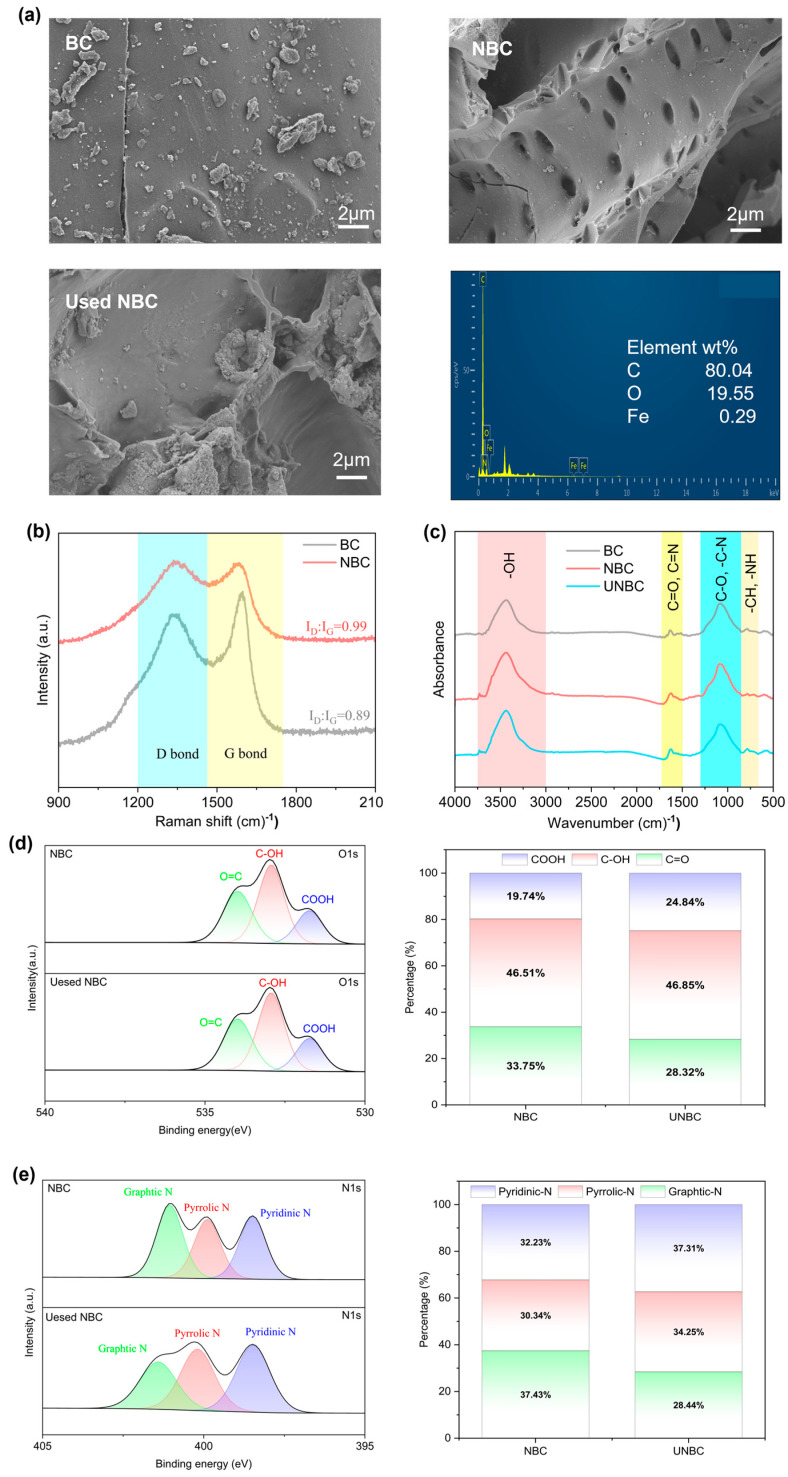
SEM-EDS image of BC, NBC and used NBC (**a**); Raman of BC and NBC (**b**); FTIR of BC and NBC (**c**); O1s (**d**) and N1s (**e**) spectra of BC and NBC.

Characterization analyses provided insights into both the morphological and structural properties of the biochar samples. The morphological properties of corn stalk biochar (BC), nitrogen-doped corn stalk biochar (NBC) and used nitrogen-doped corn stalk biochar (UNBC) were examined by scanning electron microscopy. As shown in Figure 1a, BC exhibited a relatively smooth surface with few visible pores, whereas NBC displayed abundant and heterogeneously distributed pore structures, forming a layered porous network that favors the exposure of additional active sites. Only 0.29% of Fe in the used NBC was detected, indicating that ferrate was essentially not loaded onto the carbon in the reaction system.

Raman spectroscopy was employed to evaluate the graphitization and defect density of biochar samples, with the intensity ratio of D and G bands (ID/IG) serving as an indicator of disorder in carbon materials [18]. The G band (1580 cm^−1^) reflected the stretching vibration of C-C bonds, associated with sp^2^ hybridized carbon atoms. The D band (1350 cm^−1^) related to defects and disordered structures, reflecting noncovalent interactions between carbon atoms [19]. As shown in Figure 1b, NBC exhibited a higher ID/IG ratio (0.99) than BC (0.89), suggesting a greater abundance of structural defects, which may be attributed to lattice distortion induced by nitrogen doping [20].

Fourier transform infrared (FTIR) spectroscopy was conducted on BC, NBC, and UNBC to examine changes in surface functional groups. Four characteristic peaks were observed in the FTIR spectrum (Figure 1c), located at 3447 cm^−1^ (-OH), 1623 cm^−1^ (C=O and C=N), 1092 cm^−1^ (C-O and C-N) and 796 cm^−1^ (C-H and N-H). Compared to BC, these peaks in NBC are significantly enhanced, which suggested that the nitrogen-containing functional groups had been successfully loaded onto BC. The introduction of nitrogen had exposed more oxygen-containing functional groups, consistent with the SEM results. The peak intensity of UNBC decreased slightly, while the peak position remained unchanged. This result showed that these functional groups were involved in the activation reaction, but under electrification, some functional groups were regenerated. This discovery demonstrates the stability and renewability of NBC.

XPS analysis was applied to identify the elemental composition and chemical structure of NBC and UNBC. As shown in Figure 1d,e, high-resolution spectra were collected for the O 1s and N 1s regions. To accentuate variations in the distribution of O and N species, the relative proportions of O and N components (R_N species_ and R_O species_) in NBC and UNBC were calculated separately. There are three characteristic peaks in the O1s spectra (Figure 1d) including 531.73 eV, 532.93 eV and 533.98 eV, corresponding to COOH, C-OH, and C=O groups, respectively [21]. The ratio of C=O decreased from 33.75% to 28.32%, while the ratio of COOH rose from 19.74% to 24.84%, suggesting that C=O may have accepted electrons and been partially converted to C–O during the activation process. Because C=O sites generally exhibit faster electron transfer properties, such redox transitions likely facilitate the catalytic reaction. Figure 1e shows that the high-resolution XPS spectra of N1s for NBC and UNBC could be resolved into three characteristic peaks: 398.40 eV, 399.81 eV and 400.97 eV, corresponding to pyridine N, pyrrolic N and graphitic N [22]. After NBC usage, the graphite N ratio decreased from 37.43% to 28.44%, while pyrrole N and pyridine N increased from 30.34% and 32.23% to 34.25% and 37.31%, respectively. Previous studies comparing the N ratio changes under different modification conditions showed that modified carbons with a higher graphitic N content exhibited superior catalytic activity. In this study, the ratio changes in the three N species before and after the reaction were compared, yielding consistent results that confirmed graphitic N played a dominant role in the catalysis. This indicated that graphitic N served as an active site [22]. Graphite N could alter the electronic distribution of carbon, accelerating electron transfer and thereby enhancing the electrical conductivity of carbon materials [23]. Furthermore, it could enhance the production of ROS [23].

### 2.2. Removal Efficiency of SMX in Various Systems

**Figure 2:**
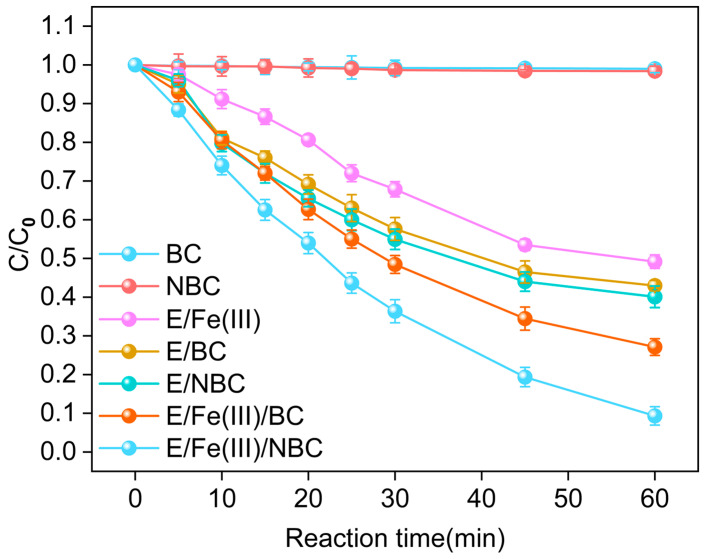
The removal rates of SMX in single system (BC, NBC), binary system (E/Fe(III), E/BC, E/NBC) and ternary system (E/Fe(III)/BC, E/Fe(III)/NBC). (C_0_ (SMX) = 0.04 mM, C_0_ (Fe^3+^) = 25 μM, E_0_ = 15 V, m (BC, NBC) = 30 mg, V = 0.05 L, t = 0–60 min.)

Single (BC, NBC), binary (E/Fe(III), E/BC and E/NBC) and ternary (E/Fe(III)/BC and E/Fe(III)/NBC) systems were constructed to compare their removal performance for SMX. As shown in Figure 2, the removal rates of SMX in BC and NBC systems were nearly zero. Due to the poor specific surface area of BC and NBC, they exhibited negligible adsorption of SMX [24]. The removal rates of SMX by E/Fe(III), E/BC and E/NBC were 50.83%, 55.72% and 67.25%. Compared with BC, the superior removal performance of NBC for SMX was attributed to the exposure of more oxygen-containing functional groups, which increased the amounts of reactive oxygen species (ROS) under electrification. The concentrations of Fe(IV)/Fe(V) were quantified using PMSO as a probe compound in the E/Fe(III) system. Section 2.6 shows that the Fe(IV)/Fe(V) concentrations were 4.2144 and 5.3488 μM at 30 and 60 min, respectively. This result suggested that Fe(III) was successfully oxidized to high-valent iron species during the reaction. Within the first 30 min, 16.85% of Fe(III) was converted to Fe(IV)/Fe(V); however, in the subsequent 30 min, only 4.53% of Fe(III) underwent this conversion. This indicated that under conditions without catalyst addition, the formation of high-valent iron species primarily occurred within the initial 30 min.

Obviously, the ternary systems showed the better removal performance for SMX, with the removal rates reaching 72.89% and 90.67% in E/Fe(III)/BC and E/Fe(III)/NBC. Moreover, the catalytic effect of NBC was remarkably significant. The addition of NBC increased the SMX removal rate from 50.83% to 90.67% within 60 min for the E/Fe(III) system.

Because of the superior electron distribution, greater defect density, and abundant functional groups of NBC, the E/Fe(III)/NBC system performed a superior degradation efficiency compared with previous studies (e.g., PMS/Mesoporous carbon (CMK)/Layered doubled hydroxides (LDHs) system achieves 84.5% within 120 min [25]). Additionally, it offers advantages such as no secondary pollution (no external oxidants required, no metal leaching from catalysts) and low operational costs (low energy consumption, high recyclability).

### 2.3. Influence of Reaction Conditions

**Figure 3:**
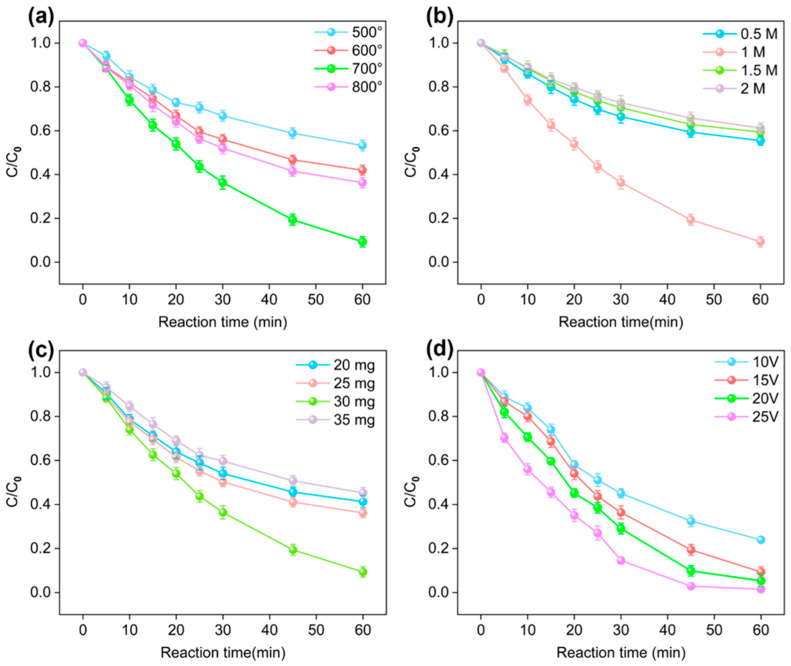
Removal efficiency of sulfamethoxazole at (**a**) different temperatures, (**b**) different concentrations of urea, (**c**) different dosages of NBC and (**d**) different input voltages. (C_0_ (SMX) = 0.04 mM, C_0_ (Fe^3+^) = 25 μM, V = 0.05 L, t = 0–60 min.)

The effects of the pyrolysis temperature, urea dosages, NBC dosage and applied voltage on the degradation rate of SMX in the E/Fe(III)/NBC system are presented in Figure 3. Figure 3a reveals that the degradation efficiencies at 500 °C, 600 °C, 700 °C, and 800 °C after 60 min were 46.73%, 58.01%, 90.67%, and 63.72%, respectively. The catalytic activity improved as the pyrolysis temperature increased from 500 °C to 700 °C. This phenomenon was attributed to the high temperatures causing damage to the carbon framework, inducing a more disordered structure and generating structural defects [26]. These changes enhanced electrical conductivity and exposed more active sites. However, as the temperature was increased from 700 °C to 800 °C, the catalytic effect decreased. This was attributed to as the pyrolysis temperature rose, the ash content also increased. For nitrogen-modified biochar, this led to a reduction in nitrogen content [27]. Evidently, pyrolysis temperatures that were too high or too low would both be detrimental to the catalytic effect of NBC. Thus, 700 °C was selected as the pyrolysis temperature for NBC.

Figure 3b describes the effect of NBC on the catalytic activity at different urea dosages. Nitrogen-modified NBC at urea concentrations of 0.5 M, 1 M, 1.5 M, and 2 M exhibited SMX degradation rates of 44.54%, 90.67%, 40.67%, and 38.81% within 60 min, respectively. Evidently, only the 1 M urea modification resulted in a marked catalytic improvement, while NBC prepared at other concentrations showed limited activity. This outcome can be attributed to two factors: (1) at low urea concentrations, insufficient nitrogen incorporation leads to fewer active sites, and (2) at excessively high concentrations, excessive urea may deposit on the carbon surface, blocking active sites and hindering catalytic performance. As shown in Figure 3c, the effect of different NBC doses on the catalytic performance was investigated. Within 60 min, the degradation rates of SMX were 58.76%, 63.85%, 90.67%, and 54.72%, respectively. Obviously, as the NBC doses increased from 10 to 30 mg, the SMX removal efficiency was also increased. This indicates that within a certain range, higher doses of NBC could provide more active sites and exhibit superior catalytic performance, such as generating more ^1^O_2_ (chapter 2.5) and Fe(IV)/Fe(V) (chapter 2.6). However, as the NBC doses increased from 30 to 35 mg, the removal efficiency of SMX decreased. It was attributed to: (1) catalyst agglomeration, which led to the coverage of active sites; and (2) self-quenching of ROS [28]. Hence, 30 mg of NBC was selected for subsequent experiments.

Obviously, increasing the applied voltage enhanced the degradation efficiency of SMX in the E/Fe(III)/NBC system, and its degradation rates at the voltages of 10, 15, 20 and 25 V were 76.10%, 90.67%, 94.68% and 98.49%, respectively (Figure 3d). Higher applied voltages accelerated the electron transfer from Fe(III), thereby promoting the formation of Fe(IV)/Fe(V). Furthermore, elevated voltages could regenerate oxygen-containing functional groups consumed on the NBC surface, such as C=O, -COOH, and -OH. Notably, the degradation rates of SMX within 60 min showed no significant differences at voltages of 10, 15, and 25V. To reduce the economic costs while achieving a high degradation rate for SMX, 15 V was selected as the optimal operating voltage for subsequent experiments.

To better estimate the economic viability of the E/Fe(III)/NBC system, energy consumption was evaluated using energy per order (E_EO_) (Equation (12)). The EEO of the E/Fe(III)/NBC system was 3.20 kWh/m^3^/order, while the EEO of the E/Fe(III) system was 10.96 kWh/m^3^/order. Obviously, the E/Fe(III)/NBC system exhibited lower energy consumption.

(12) $$E_{\mathrm{EO}}=\frac{\mathrm{UIt}}{\mathrm{Vlog}(\frac{C0}{\mathrm{Ct}})}$$

where U is the voltage (V) and I is the current (A). t is the operating time (h), and V represents the volume (m^3^) of the treated solution. C_0_ and C_t_ represent the initial and residual concentrations of the SMX, respectively.

### 2.4. Effect of Water Chemistry

Natural water bodies typically contain various inorganic anions and organic matter, which could inhibit the degradation of SMX. Different concentrations (5–10 mM) of Cl^−^, NO^3−^, CO_3_^2−^, PO_4_^3−^ and HCO_3_^−^ were carried out to study their effects on SMX degradation by the E/Fe(III)/NBC system. Specifically, Cl^−^ exhibited a significant catalytic effect on the degradation of SMX. When the Cl^−^ concentration reached 10 mM, complete removal of all SMX occurred within just 15 min. This enhancement can be attributed to the generation of reactive chlorine species through an electrooxidation process (Equations (13) and (14)), as well as the fact that Cl^−^ does not significantly compete with SMX for ROS and Fe(IV)/Fe(V) [29].

**Figure 4:**
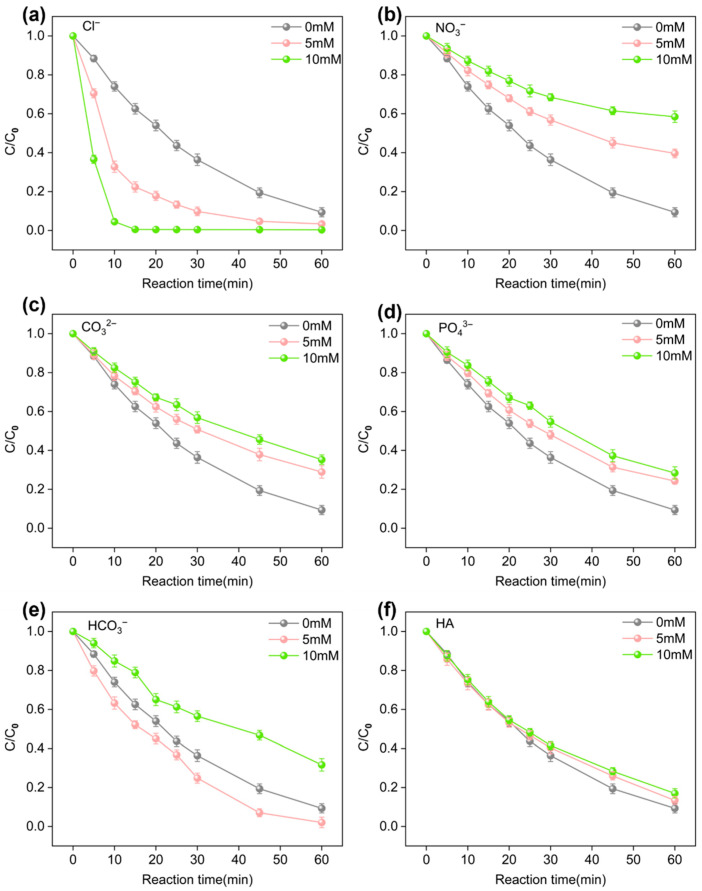
Effect of Cl^−^ (**a**), NO^3−^ (**b**), CO_3_^2−^ (**c**), PO_4_^3−^ (**d**), HCO_3_^−^ (**e**), HA (**f**) concentrations on SMX degradation in EC/Fe(III)/NBC system. (C_0_ (SMX) = 0.04 mM, C_0_ (Fe^3+^) = 25 μM, E_0_ = 15 V, m (NBC) = 30 mg, V = 0.05 L, t = 0–60 min.)

It could be observed that different concentrations of NO_3_^−^, CO_3_^2−^, and PO_4_^3−^ ions exhibited varying inhibitory effects on SMX removal (Figure 4b–d). The order of the ionic inhibition of SMX removal was NO_3_^−^(41.55%) > CO_3_^2−^(64.76%) > PO_4_^3−^(71.63%) at a concentration of 10 mM. Excessive NO_3_^−^ and CO_3_^2−^ would quench ROS [30], such as ·OH, generating species with lower oxidation capacities such as NO_3_· and CO_3_^−^·(Equations (15)–(18)). PO_4_^3−^, on the other hand, could form complexes with Fe(V) and reduce its redox potential [31], thereby influencing its reaction with SMX. PO_4_^3−^ complexes with Fe(V) may induce steric hindrance, limiting the binding of Fe(V) complexes to target pollutions and thus affecting electron transfer processes [28].

Different concentrations of HCO_3_^−^ exhibited opposite effects on SMX removal (Figure 4). Lower concentrations (5 mM) of HCO_3_^−^ slightly promoted SMX degradation, increasing the removal rate to 97.94%, whereas higher concentrations (10 mM) of HCO_3_^−^ exhibited an inhibitory effect, reducing the removal rate to 68.38%. It was very likely that low concentrations of HCO_3_^−^ reduced the redox potential and prolonged the lifetime of Fe(V) by complexing with Fe(V) [32]. This prevented the rapid spontaneous self-decomposition of Fe(V) and promoted the oxidation of SMX. High concentrations of HCO_3_^−^ demonstrated ROS scavenging properties, which exerted a significant influence on reactions primarily governed by ROS (Equation (19)) [33].

(13) $${\mathrm{Cl}}^{-}\to \cdot \mathrm{Cl}+e^{-}$$

(14) $$\cdot \mathrm{Cl}+\cdot \mathrm{Cl}\to \cdot {\mathrm{Cl}}_{2}$$

(15) $${\mathrm{NO}}_{3^{-}}\to {\mathrm{NO}}_{3}\cdot +e^{-}$$

(16) $${\mathrm{NO}}_{3^{-}}\;+\cdot \mathrm{OH}\to {\mathrm{NO}}_{3}\cdot +{\mathrm{OH}}^{-}$$

(17) $${\mathrm{CO}}_{3^{2-}}\to {\mathrm{CO}}_{3^{-}}\cdot +e^{-}$$

(18) $${\mathrm{CO}}_{3^{2-}}+\cdot \mathrm{OH}\to {\mathrm{CO}}_{3^{-}}\cdot +{\mathrm{OH}}^{-}$$

(19) $${\mathrm{HCO}}_{3^{-}}+\cdot \mathrm{OH}\to {\mathrm{CO}}_{3^{-}}\cdot +H_{2}O$$

As shown in Figure 4f, HA had a less inhibitory effect on SMX degradation, with the removal rate decreasing from 90.7% to 82.99%. This was because HA competed with SMX for ROS, but its reactivity with ROS was lower than that of SMX, resulting in a smaller impact.

### 2.5. Identification of the Dominant ROS and Electron Transfer

#### 2.5.1. Identification of the Dominant ROS and Mechanism

**Figure 5:**
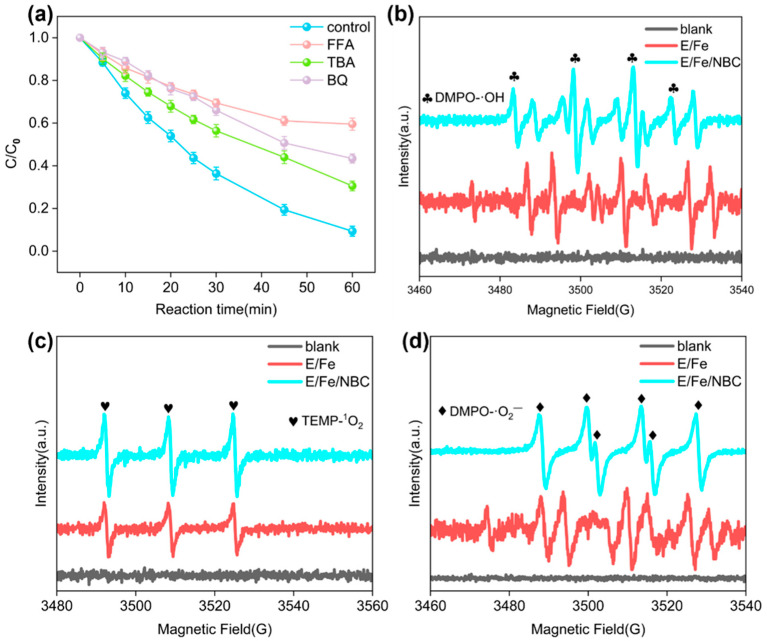
Effects of different quenchers on SMX degradation efficiency (**a**); EPR spectra for ·OH, ^1^O_2_ and ·O_2_^−^ (**b**–**d**).

To investigate the roles of reactive oxygen species (ROS) in the E/Fe(III)/NBC system, a series of quenching experiments, EPR analysis, and probe tests were carried out. Amounts of 10 mM furfuryl alcohol (FFA), tert-butyl alcohol (TBA), and p-benzoquinone (p-BQ) were employed to quench ^1^O_2_, ·OH and ·O_2_^−^ in the E/Fe(III)/NBC system, respectively. The aim was to evaluate the contribution of each species to SMX degradation. All three quenchers performed various degrees of inhibition on SMX removal. The removal efficiencies of FFA, TBA, and BQ for SMX were 40.5%, 69.46%, and 56.63% (Figure 5a). These results indicated that ^1^O_2_, ·OH and ·O_2_^−^ all participated in SMX degradation. ·O_2_^−^ was the precursor to ^1^O_2_ [16] and ·OH was used to synthesize Fe(V) (Equations (3) and (4)). The contribution to SMX degradation, from highest to lowest, was ^1^O_2_> ·O_2_^−^> ·OH. This meant that ^1^O_2_ may be one of the primary non-radical species in the system.

EPR experiments further validated the ROS generated in the E/Fe(III)/NBC system. DMPO and TEMP were employed as spin-trapping agents for capturing ·OH/·O_2_^−^ and ^1^O_2_ at 20 min, respectively. As shown in Figure 5b–d, the peak signal for ^1^O_2_ was relatively prominent, while those for ·OH and ·O_2_^−^ were comparatively weak in the E/Fe(III) system. Notably, the peak signals for ^1^O_2_, ·OH, and ·O_2_^−^ significantly increased after adding NBC. Specifically, the ^1^O_2_ signal in the E/Fe(III)/NBC system was approximately 2.5 times higher than that observed in the E/Fe(III) system. Previous studies demonstrated that functional groups on the surface of NBC (e.g., C=O) could react with Fe(IV)/Fe(V) to generate ^1^O_2_ [34]. FTIR and XPS analysis showed that under electrically charged conditions, which promoted the regeneration of functional groups on the NBC surface, the C=O peak signal and ratio remained reduced. This indicated that C=O served as the catalytic center for ^1^O_2_ formation [35]. On the other hand, the decomposition of Fe(IV)/Fe(V) first produced H_2_O_2_, and further generated ^1^O_2_ (Equations (20)–(22)) [36]. H_2_O_2_ also accepted electrons from EC or oxygen-containing functional groups, accompanied by the generation of ·OH and ·O_2_^−^. In summary, the addition of NBC significantly benefited ROS production while substantially increasing the concentration of Fe(IV)/Fe(V).

(20) $$\mathrm{Fe}(V)+2H_{2}O+2H^{+}\to 2\mathrm{Fe}(\mathrm{III})+2H_{2}O_{2}$$

(21) $$2\mathrm{Fe}\left(\mathrm{IV}\right)\to 2\mathrm{Fe}(\mathrm{III})+\frac{1}{3}H_{2}O+\frac{1}{3}H_{2}O_{2}$$

(22) Fe(V)+H2O2+2H+→Fe(III)+O21+H2O

#### 2.5.2. Electron Transfer

Chronoamperometry (i-t) and linear sweep voltammetry (LSV) were performed to compare the electron transfer behavior of BC and NBC (Figure S1). It was clear that the addition of Fe(III) resulted in a marked increase in the current for both the BC and NBC, with NBC exhibiting a much greater enhancement. (Figure S1a). This indicated that NBC facilitated a more efficient electron transfer to Fe(III). The addition of SMX resulted in a transient increase in the current observed in the E/Fe(III)/NBC system, which promptly returned to its original state. However, this response was relatively minor in the E/Fe(III)/BC system. This result demonstrated that the Fe(IV)/Fe(V) interacted with SMX to form transient reaction products, temporarily disrupting the system equilibrium [37]. LSV measurements were further used to evaluate the influence of BC and NBC on the electron transfer rates in the E/Fe(III)/SMX system (Figure S1b). As the voltage increased, the current intensity gradually rose, with the NBC curve exhibiting a steeper upward trend than the BC curve. In the E/Fe(III)/SMX system, biochar served as a conductive bridge [38], and the NBC curve demonstrated a steeper slope. This revealed that the response current of NBC was higher than that of BC, demonstrating NBC’s superior electrical conductivity. Combined with the results above, biochar (BC and NBC), Fe(III), and SMX directly exhibited electron transfer, with NBC demonstrating superior catalytic ability to BC in promoting electron migration efficiency among these three components.

### 2.6. Identification of Fe(IV)/Fe(V)

**Figure 6:**
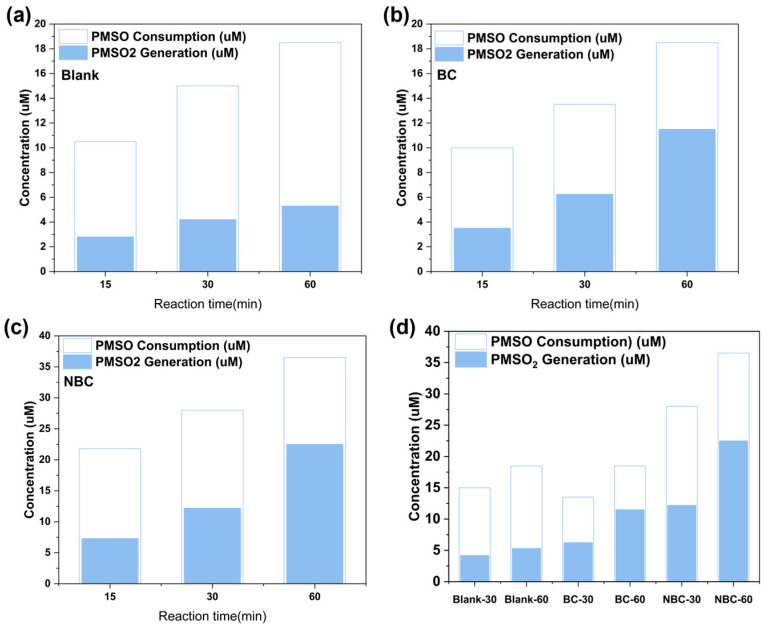
Degradation of PMSO and generation of PMSO_2_ in different systems ((**a**) Blank; (**b**) BC; (**c**) NBC; (**d**) Comparison.). (C_0_ (SMX) = 0.04 mM, C_0_ (Fe^3+^) = 25 μM, E_0_ = 15 V, m (BC, NBC) = 30 mg, C_0_ (PMSO) = 50 μM, V = 0.05 L, t = 0–60 min.)

Even after quenching ^1^O_2_, the E/Fe(III)/NBC system still exhibited a 40.5% removal rate for SMX, indicating that Fe(IV)/Fe(V) also played a significant role in SMX degradation. Because Fe(IV)/Fe(V) can oxidize methyl phenyl sulfoxide (PMSO) to methyl phenyl sulfone (PMSO_2_), PMSO was used as a probe to quantify high-valent iron species in the three systems (E/Fe(III), E/Fe(III)/BC, and E/Fe(III)/NBC, Figure 6). Obviously, in all three systems, the consumption of PMSO exceeded its production of PMSO_2_, because ROS also oxidized PMSO without generating PMSO_2_. Instead, they led to the formation of hydroxylated or polymerized products [39]. The consumption of PMSO was 15 μM and 18.5 μM, respectively, while the production of PMSO_2_ was 4.21 μM and 5.34 μM at 30 and 60 min in the E/Fe(III) system. This result showed that, consistent with previous in situ electrochemically generating Fe(IV)/Fe(V) systems, Fe(IV)/Fe(V) were primarily generated within the first 30 min. The consumption of PMSO was 13.03 μM and 18.04 μM at 30 min and 60 min after adding BC, while the generation of PMSO_2_ was 6.24 μM and 11.50 μM. It could be observed that the generation rate of Fe(IV)/Fe(V) remained relatively stable during the first 30 min and the subsequent 30 min. This stability was attributed to the promotion of ·OH generation by the C=C on BC [40], thereby enhancing the production of Fe(IV)/Fe(V) (Equations (3) and (4)). Based on this, the formation of Fe(IV)/Fe(V) in the E/Fe(III)/NBC system was investigated. At 30 and 60 min, the consumption of PMSO was 27.95 μM and 36.68 μM, respectively, while the generation of PMSO_2_ was 12.12 μM and 22.35 μM. This result was attributed to nitrogen atom doping causing deformation of the carbon layer, thereby creating more defect sites [27].

### 2.7. Stability and Renewability of NBC

This study evaluated the stability and reusability of NBC in the E/Fe(III)/NBC system through cyclic experiments. Figure S2 illustrates that E/Fe(III)/NBC maintained excellent sustainable removal performance for SMX during reuse cycles. After five cycles, the SMX removal rate remained as high as 66.69%, with a decrease of only 23.02%. This decrease may result from degradation intermediates covering active sites, reducing the graphitic nitrogen content and diminishing the catalytic efficiency. However, the electrochemical treatment introduced in this study provided sufficient electrons to regenerate oxygen-containing functional groups on the NBC surface (e.g., -COO• and -O•), in order to maintain its sustainable catalytic performance.

### 2.8. Performance of E/Fe(III)/NBC System in Actual Aquatic Environment and SA Degradation Performance

To further test the applicability of the E/Fe(III)/NBC system in real aquatic environments, three types of real water samples (tap water, wastewater, and Qiantang River water) were used in the SMX removal experiments. As shown in Figure S3a, the results indicated that 88.93%, 77.6%, and 82.56% of SMX were removed in the E/Fe(III)/NBC system within 60 min, respectively. In tap water and river water, various organic compounds and inorganic ions (e.g., bicarbonate) may participate in competitive reactions, consuming ·OH and potentially limiting the oxidation efficiency. Meanwhile, hypochlorous acid present in tap water can act as an oxidant for organic pollutants, thereby enhancing SMX degradation [41]. The abundant organic matter and microorganisms in wastewater may consume the active species within the E/Fe(III)/NBC system, leading to a reduced removal efficiency. Overall, the E/Fe(III)/NBC system demonstrates broad prospects and practical application value for SMX treatment in real aquatic environments.

The degradation performance of the E/Fe(III)/NBC system toward other sulfonamide antibiotics was investigated to assess the system’s applicability. Different types of sulfonamide compounds all possess amino groups, sulfonamide bridges, and benzene moieties, while the R substituent determines the specific type of sulfonamide [42]. The degradation rates of SDZ, SMP, STZ, SDX, and SMX were 89.01%, 74.87%, 73.58%, 85.21%, and 90.07%, respectively. Compared to SMX, SMP and STZ exhibited stronger resistance to degradation. This difference may be attributed to the susceptibility of the isoxazole structure to oxidative attack, whereas thiazole and pyrimidine structures demonstrate opposite characteristics in this regard [43]. In conclusion, the E/Fe(III)/NBC system demonstrated outstanding degradation efficiency for various sulfonamide drugs, exhibiting strong practical applicability in the field of sulfonamide contamination removal.

### 2.9. Possible Degradation Pathway of SMX

**Figure 7:**
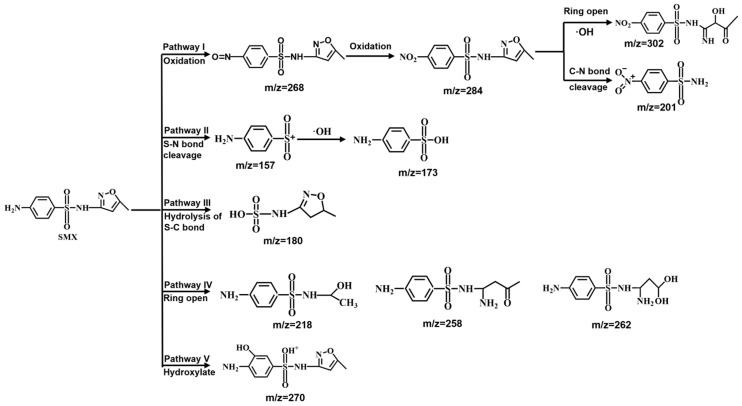
Predicted degradation pathways of SMX in E/Fe(III)/NBC system.

LC-MS analysis was conducted to identify the degradation products of SMX in the E/Fe(III)/NBC system. Based on the detected intermediates, five possible degradation pathways were proposed (Figure 7), involving oxidation, bond cleavage, hydrolysis, ring opening and hydroxylation. In pathway I, m/z = 268 and m/z = 284 were respectively formed by the one-step and two-step oxidation of the -NH_2_ group on the aromatic ring of SMX [44], and then followed by two reactions: (1) isoxazole undergoes a ring-opening reaction followed by hydroxylation to form m/z = 302 [45], and (2) the cleavage of the C-N bond resulted in the partial separation of isoxazole and the sulfonamide moiety, yielding m/z = 201. In pathway II, the S-N bond underwent cleavage to form m/z = 157, followed by hydroxylation to yield m/z = 173. In pathway III, the m/z = 180 was obtained through the hydrolysis of the S-C bond [46]. In pathway IV, the ring opening of isoxazole was also an identifiable step in the degradation mechanism. This process ultimately resulted in products with m/z = 258, m/z = 218 and m/z = 262. The process of electron transfer from iron atoms to isoxazole had been identified as the critical stage in isoxazole cleavage during Fe(V) oxidation. In pathway V, SMX was hydrolyzed, and then obtained m/z = 270.

### 2.10. Toxicity Assessment of Degradation Products

**Figure 8:**
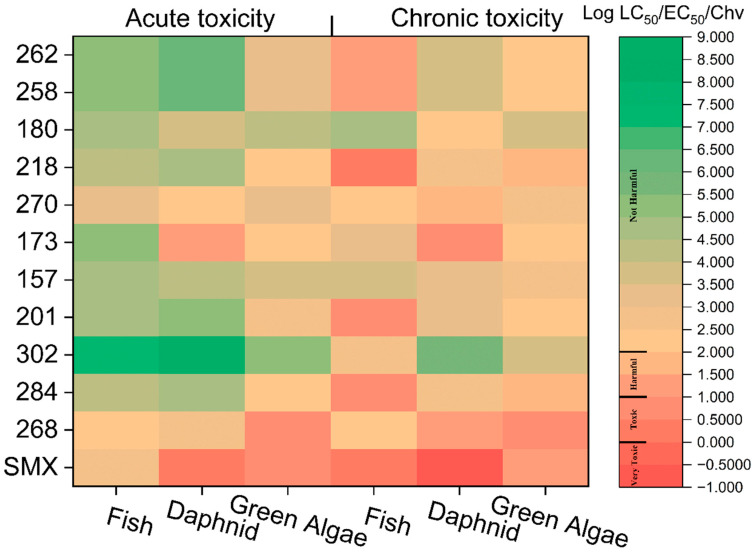
The acute and chronic toxicity assessment of SMX and its TPs.

Ecotoxicity assessment is considered one of the key evaluation metrics for determining the application potential of the E/Fe(III)/NBC system in SMX removal. The ECOSAR 2.0 model was employed to simulate the acute toxicity (LC50) and chronic toxicity (Ch V) of SMX degradation intermediates on fish, Daphnia magna, and green algae (Figure 8). According to the Globally Harmonized System (GHS), the toxicity classifications for SMX and its intermediates fall into four categories: highly toxic, toxic, harmful, and harmless. Among acute toxicity assessments, SMX was toxic to green algae and Daphnia but harmless to fish. Degradation products presented significantly lower toxicity to fish, Daphnia, and green algae relative to SMX. Similar results were observed in chronic toxicity predictions, where SMX exhibited high toxicity to fish, Daphnia, and green algae, while other degradation products showed no adverse effects on these organisms. As a whole, the degradation of SMX by the E/Fe(III)/NBC system significantly reduced the aquatic toxicity of SMX by generating less toxic TPs, showing that this system had a favorable detoxification effect on SMX.

### 2.11. Theoretical Calculations

**Figure 9:**
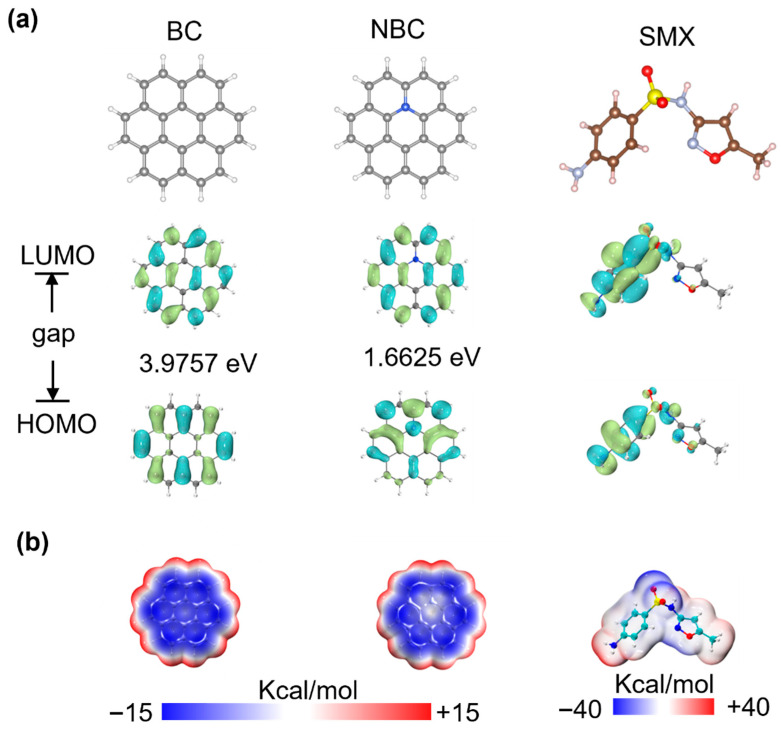
The optimized chemical structure, LUMO, HOMO of BC, NBC and SMX (**a**); distribution of electrostatic potential (ESP) of BC, NBC and SMX (**b**).

DFT calculations were employed to investigate the catalytic mechanism of biochar and the degradation mechanism of SMX for a deeper understanding. In this study, eight carbon cluster models of biochar were used to represent the biochar structure. Given that graphite N served as the primary active site, the N atom doping position was exemplified by graphite N as the subject of study. Figure 9 displays the optimized molecular structures, highest occupied molecular orbitals (HOMOs), lowest unoccupied molecular orbitals (LUMOs) and electrostatic potential (ESP) distributions for BC, NBC and SMX. Molecular orbital theory, particularly the lowest unoccupied molecular orbital (LUMO) and highest occupied molecular orbital (HOMO), provides insights into reactivity [47]. Compared to BC, NBC had a narrower HOMO-LUMO gap. The reduced bandgap indicated that less energy was required for excitation. A narrower gap allowed active electrons to migrate more readily across its surface, thereby promoting the formation of high-iron species [48]. Electrostatic potential (ESP) provided an electron distribution, where regions with pronounced positive or negative potentials are more probable to participate in redox reactions [49]. The N atoms on the NBC surface had been strategically rearranged to alter their electronic structure, exposing more active sites and carbon defect sites that could serve as catalytic centers [50]. The higher electron density on the aniline ring of SMX indicated that this portion may be readily susceptible to attack by reactive species (Figure 9a) [51]. The results revealed that the -NH_2_ group was highly distributed in the HOMO region, which enhanced its sensitivity to electrophilic attacks from reactive species and electrophiles such as ^1^O_2_ and Fe(IV)/Fe(V) [52]. The corresponding products were identified as m/z = 268 and m/z = 284. ESP (Figure 9b) suggested that the regions surrounding the S-N bond in the sulfonamide moiety presented a high electron density, indicating susceptibility to attack by electrophilic reagents [53]. The most negatively charged sulfonamide moiety was easily attacked and reacted with the analyte in the initial stage, yielding corresponding products at m/z = 157 and m/z = 180.

## 3. Materials and Methods

### 3.1. Chemicals

Materials and chemicals employed in this work were summarized in Text S1.

### 3.2. Preparation of Biochar

Firstly, the corn stalks were smashed by a grinder with average particle sizes of around 60 meshes and dried at 105 °C for 2 h in an oven. Briefly, 6.5 g urea was completely dissolved in 100 mL pure water, and then 10 g corn stalk powder was soaked in urea solution, stirred by a magnetic stirrer for 12 h and then dried at 105 °C in an oven. It was then placed into a tube furnace for pyrolysis under the following conditions: N_2_ flow rate of 0.5 L min, temperature ramp rate of 5 °C and maintained at 700 °C for 2 h. Biochar was washed with pure water and ethanol, and dried at 60 °C in an oven. This N-doped biochar was labeled NBC.

### 3.3. Batch Experiments

All experiments were conducted in 50 mL beakers at room temperature (25 ± 2 °C) with a rotor speed of 200 rpm. The anode was carbon paper, and the cathode was platinum wire. Detailed experimental conditions were provided in the figure captions. In the electrochemical experiments, a buffered SMX solution of pH 9.0 was prepared using 0.1 M borate buffer, simultaneously added Fe_2_(SO_4_)_3_ and NBC at a voltage of 15 V. 2.0 mL of the sample, obtained at corresponding time intervals, was filtered through 0.22 μM membrane. Sodium thiosulfate (1.0 mL, 0.1 M) was immediately added to terminate the reactions. For accuracy, all experiments were repeated three times.

Concentrations of target pollutants were detected with high-performance liquid chromatography (HPLC). PMSO and PMSO_2_ concentrations were determined by HPLC. Reactive oxygen species (ROS) were identified by electron paramagnetic resonance (EPR) tests. The linear sweep voltammetry (LSV) and chronoamperometry (i-t) were detected using an electrochemical workstation (CHI660E). Intermediate products were detected by LC-MS/MS. ECOSAR was utilized to analyze the toxicity of the intermediates produced in the system. Detailed analytical methods are provided in the Supplementary Materials (Text S2–S5).

### 3.4. DFT Calculation

To describe the degradation mechanism of SMX more precisely, the degradation sites were employed using DFT quantitative calculations. The Gaussian 09 pack using a 6-31G* (d, p) (liquid phase) standard basis set was used to optimize the molecular structure of SMX. After conducting theoretical calculations, the electrostatic potentials and frontier electron orbitals were visualized using the Multiwfn 3.6 and VMD 1.9.3 software programs.

## 4. Conclusions

This study demonstrated that low concentrations of Fe(III) can be directly oxidized to Fe(IV)/Fe(V) and subsequently activated by NBC to efficiently degrade SMX. Within 60 min, 90.67% of SMX was degraded. Excessive background components (such as NO_3_^−^, CO_3_^2−^, PO_4_^3−^ and HCO_3_^−^) inhibited SMX removal, but Cl^−^ significantly promoted SMX removal. Quenching experiments and PMSO experiments demonstrate that ^1^O_2_ and Fe(IV)/Fe(V) were the primary contributors to SMX degradation. Electrochemistry not only facilitated Fe(III) oxidation but also regenerated oxygen-containing functional groups on the NBC surface, enhancing NBC stability and reusability. The E/Fe(III)/NBC system performed a high degradation efficiency on sulfonamide antibiotics in real aquatic environments. During the degradation process, SMX underwent oxidation, bond cleavage, hydrolysis, ring opening and hydroxylation. The degradation products exhibited lower predicted toxicity to fish, Daphnia, and green algae. Raman spectroscopy showed that nitrogen doping induced deformation of the carbon layers, thus exposing more active sites. XPS and DFT calculations indicated that graphitic N was the main active site, as it modulated the local charge distribution of carbon and lowered the reaction energy barrier. Above all, the E/Fe(III)/NBC system developed in this study demonstrated significant potential for effectively eliminating antibiotics in aqueous solutions without the addition of any oxidizing agents. It also provides a new approach for promoting the in situ generation of Fe(IV)/Fe(V), highlighting its strong potential for practical environmental applications.

## Data Availability

The raw data supporting the conclusions of this article will be made available by the authors upon request.

## Supplementary Materials

The following supporting information can be downloaded at: https://www.mdpi.com/article/10.3390/antibiotics15030254/s1.

## Abbreviations

SMX	sulfamethoxazole
SDZ	sulfadiazine
SDX	sulfadoxine
SMP	sulfamethoxypyridazine
STZ	sulfathiazole
PMSO	methyl phenyl sulfoxide
PMSO_2_	methyl phenyl sulfone
BC	corn stalk biochar
NBC	nitrogen-doped corn stalk biochar
UNBC	used NBC
TBA	tert-butanol
FFA	furfuryl alcohol
BQ	benzoquinone
HA	humic acid
DMPO	5,5-dimethyl-1-pyran N-oxide
TEMP	2,2,6,6-tetramethyl-4-piperidinol
ROS	reactive oxygen species
DFT	density functional theory
TPs	transformation products
AOPs	advanced oxidation processes
EPR	electron paramagnetic resonance

## References

[1] García-Galán M.J., Silvia Díaz-Cruz M., Barceló D., Barceló D. (2009). Combining Chemical Analysis and Ecotoxicity to Determine Environmental Exposure and to Assess Risk from Sulfonamides. TrAC Trends Anal. Chem..

[2] Klein E.Y., Milkowska-Shibata M., Tseng K.K., Sharland M., Gandra S., Pulcini C., Laxminarayan R. (2021). Assessment of WHO Antibiotic Consumption and Access Targets in 76 Countries, 2000–2015: An Analysis of Pharmaceutical Sales Data. Lancet Infect. Dis..

[3] Ngumba E., Gachanja A., Tuhkanen T. (2016). Occurrence of Selected Antibiotics and Antiretroviral Drugs in Nairobi River Basin, Kenya. Sci. Total Environ..

[4] Wang J., Wang S. (2018). Microbial Degradation of Sulfamethoxazole in the Environment. Appl. Microbiol. Biotechnol..

[5] Johansson C.H., Janmar L., Backhaus T. (2014). Toxicity of Ciprofloxacin and Sulfamethoxazole to Marine Periphytic Algae and Bacteria. Aquat. Toxicol..

[6] Wang Y., Yang X., Li H., Zhu L., Wang H. (2023). Steel Slag Assists Potassium Ferrate to Improve SCFAs Production from Anaerobic Sludge Fermentation. J. Environ. Manag..

[7] Du L., Ahmad S., Liu L., Wang L., Tang J. (2023). A Review of Antibiotics and Antibiotic Resistance Genes (ARGs) Adsorption by Biochar and Modified Biochar in Water. Sci. Total Environ..

[8] Wang B., Xu M., Chi C., Wang C., Meng D. (2017). Degradation of Methyl Orange Using Dielectric Barrier Discharge Water Falling Film Reactor. J. Adv. Oxid. Technol..

[9] Ding H., Deng Z., Ma Y., Xiang X., Xiang M., Zhang Z. (2025). Self-Doped Biochar with Adsorbed Aqueous Nitrogen for Efficient Activation of Peroxymonosulfate to Degrade Sulfamethoxazole. Chem. Eng. J..

[10] Wang X., Liu P., Fu M., Ma J., Ning P. (2016). Novel Sequential Process for Enhanced Dye Synergistic Degradation Based on Nano Zero-Valent Iron and Potassium Permanganate. Chemosphere.

[11] Wang S., Lin Y., Shao B., Dong H., Ma J., Guan X. (2023). Selective Removal of Emerging Organic Contaminants from Water Using Electrogenerated Fe(IV) and Fe(V) under Near-Neutral Conditions. Environ. Sci. Technol..

[12] McBeath S.T., Zhang Y., Hoffmann M.R. (2023). Novel Synthesis Pathways for Highly Oxidative Iron Species: Generation, Stability, and Treatment Applications of Ferrate(IV/V/VI). Environ. Sci. Technol..

[13] Liu B., Zhang Z., Guan D., Wang B., Zhou S., Chen T., Wang J., Li Y., Gao B. (2023). Qualitative and Quantitative Analysis for Cd2+ Removal Mechanisms by Biochar Composites from Co-Pyrolysis of Corn Straw and Fly Ash. Chemosphere.

[14] Duan X., Sun H., Wang Y., Kang J., Wang S. (2015). N-Doping-Induced Nonradical Reaction on Single-Walled Carbon Nanotubes for Catalytic Phenol Oxidation. ACS Catal..

[15] Ding D., Yang S., Qian X., Chen L., Cai T. (2020). Nitrogen-Doping Positively Whilst Sulfur-Doping Negatively Affect the Catalytic Activity of Biochar for the Degradation of Organic Contaminant. Appl. Catal. B Environ..

[16] Zhang K., Yi Y., Fang Z. (2023). Remediation of Cadmium or Arsenic Contaminated Water and Soil by Modified Biochar: A Review. Chemosphere.

[17] Wang W., Chen M. (2022). Catalytic Degradation of Sulfamethoxazole by Peroxymonosulfate Activation System Composed of Nitrogen-Doped Biochar from Pomelo Peel: Important Roles of Defects and Nitrogen, and Detoxification of Intermediates. J. Colloid Interface Sci..

[18] Ma Y., Tang J., Chen S., Yang L., Shen S., Chen X., Zhang Z. (2023). Ball Milling and Acetic Acid Co-Modified Sludge Biochar Enhanced by Electrochemistry to Activate Peroxymonosulfate for Sustainable Degradation of Environmental Concentration Neonicotinoids. J. Hazard. Mater..

[19] Zhang Y., Wang T., Zhang X., Sun Y., Fan G., Song G., Chai B. (2024). Porous Pie-like Nitrogen-Doped Biochar as a Metal-Free Peroxymonosulfate Activator for Sulfamethoxazole Degradation: Performance, DFT Calculation and Mechanism. Appl. Surf. Sci..

[20] Li D., Duan X., Sun H., Kang J., Zhang H., Tade M.O., Wang S. (2017). Facile Synthesis of Nitrogen-Doped Graphene via Low-Temperature Pyrolysis: The Effects of Precursors and Annealing Ambience on Metal-Free Catalytic Oxidation. Carbon.

[21] Wang L., Jiang S., Huang J., Jiang H. (2022). Oxygen-Doped Biochar for the Activation of Ferrate for the Highly Efficient Degradation of Sulfadiazine with a Distinct Pathway. J. Environ. Chem. Eng..

[22] Jingjing Y., Jinling W., Hong L., Yurong D., Chen Y., Qing Z., Zhi D. (2022). Nitrogen-Doped Biochar as Peroxymonosulfate Activator to Degrade 2,4-Dichlorophenol: Preparation, Properties and Structure–Activity Relationship. J. Hazard. Mater..

[23] He D., Zhu K., Huang J., Shen Y., Lei L., He H., Chen W. (2022). N, S Co-Doped Magnetic Mesoporous Carbon Nanosheets for Activating Peroxymonosulfate to Rapidly Degrade Tetracycline: Synergistic Effect and Mechanism. J. Hazard. Mater..

[24] Przepiórski J., Karolczyk J., Takeda K., Tsumura T., Toyoda M., Morawski A.W. (2009). Porous Carbon Obtained by Carbonization of PET Mixed with Basic Magnesium Carbonate: Pore Structure and Pore Creation Mechanism. Ind. Eng. Chem. Res..

[25] Wang Z., Li Y., Shen G., Li Y., Zhang X., Gou J., Cheng X. (2021). Synthesis of CMK/LDH and CMK/CLDH for Sulfamethoxazole Degradation by PS Activation: A Comparative Study of Characterization and Operating Parameter, Mechanism Pathway. Sep. Purif. Technol..

[26] Li Z., Sun Y., Yang Y., Han Y., Wang T., Chen J., Tsang D.C.W. (2020). Biochar-Supported Nanoscale Zero-Valent Iron as an Efficient Catalyst for Organic Degradation in Groundwater. J. Hazard. Mater..

[27] Leng L., Xu S., Liu R., Yu T., Zhuo X., Leng S., Xiong Q., Huang H. (2020). Nitrogen Containing Functional Groups of Biochar: An Overview. Bioresour. Technol..

[28] Jiang T., Wang B., Gao B., Cheng N., Feng Q., Chen M., Wang S. (2023). Degradation of Organic Pollutants from Water by Biochar-Assisted Advanced Oxidation Processes: Mechanisms and Applications. J. Hazard. Mater..

[29] Zeng C., Ma Y., Zhou Y., Chen X., Deng Z., Liu Y., Zhang Z. (2025). In-Situ Electrochemically Co-Generating Fe(IV), •OH, and SO4•- for Antibiotics Degradation in Waters. J. Environ. Chem. Eng..

[30] He H., Zhao J. (2023). The Efficient Degradation of Diclofenac by Ferrate and Peroxymonosulfate: Performances, Mechanisms, and Toxicity Assessment. Env. Sci. Pollut. Res..

[31] Huang Z.-S., Wang L., Liu Y.-L., Jiang J., Xue M., Xu C.-B., Zhen Y.-F., Wang Y.-C., Ma J. (2018). Impact of Phosphate on Ferrate Oxidation of Organic Compounds: An Underestimated Oxidant. Environ. Sci. Technol..

[32] Luo C., Feng M., Sharma V.K., Huang C. (2019). Oxidation of Pharmaceuticals by Ferrate(VI) in Hydrolyzed Urine: Effects of Major Inorganic Constituents. Environ. Sci. Technol..

[33] Ma J., Graham N.J.D. (2000). Degradation of Atrazine by Manganese-Catalysed Ozonation—Influence of Radical Scavengers. Water Res..

[34] Pan B., Feng M., McDonald T.J., Manoli K., Wang C., Huang C., Sharma V.K. (2020). Enhanced Ferrate(VI) Oxidation of Micropollutants in Water by Carbonaceous Materials: Elucidating Surface Functionality. Chem. Eng. J..

[35] Deng Z., Zhu J., Zeng C., Mu R., Ma Y., Zhang Z. (2024). Highly Efficient Activation of Ferrate (VI) via Corncob Biochar Assisted by Electrochemistry for the Removal of Sulfamethoxazole from Water. Chem. Eng. J..

[36] Shao B., Dong H., Sun B., Guan X. (2019). Role of Ferrate(IV) and Ferrate(V) in Activating Ferrate(VI) by Calcium Sulfite for Enhanced Oxidation of Organic Contaminants. Environ. Sci. Technol..

[37] Deng Z., Ma Y., Zhu J., Zeng C., Mu R., Liu Y., Li P., Zhang Z. (2024). Novel Insights into Ferrate (VI) Activation by Mn-Modified Sludge Biochar for Sulfamethoxazole Degradation: Dominance of Hydroxyl Group and Mn-O Bond in the Non-Radical Pathway. Sep. Purif. Technol..

[38] Chu D., Dong H., Li Y., Jin Z., Xiao J., Xiang S., Dong Q., Hou X. (2022). Enhanced Activation of Sulfite by a Mixture of Zero-Valent Fe-Mn Bimetallic Nanoparticles and Biochar for Degradation of Sulfamethazine in Water. Sep. Purif. Technol..

[39] Cao J., Du Y., Dai X., Liu T., Wang Z., Li J., Zhang H., Zhou P., Lai B. (2024). Ferrate(VI)-Based Synergistic Oxidation Processes (Fe(VI)-SOPs): Promoted Reactive Species Production, Micropollutant/Microorganism Elimination, and Toxicity Reduction. Chem. Eng. J..

[40] Zhang H., Li L., Liu X., Zhang X., Liu X., Dong L., Li P., Xue M., Li B., Xia G. (2024). Critical Impact of Biochar on Hydroxyl Radical Generation during Humin Oxidation. Chem. Eng. J..

[41] Liu Z., Zhao C., Wang P., Zheng H., Sun Y., Dionysiou D.D. (2018). Removal of Carbamazepine in Water by Electro-Activated Carbon Fiber-Peroxydisulfate: Comparison, Optimization, Recycle, and Mechanism Study. Chem. Eng. J..

[42] Cui S., Qi Y., Zhu Q., Wang C., Sun H. (2023). A Review of the Influence of Soil Minerals and Organic Matter on the Migration and Transformation of Sulfonamides. Sci. Total Environ..

[43] Zeng X., Wang X., Shu S., Zhang R., Chen J., Wang Y. (2025). Constructing a Z-Scheme β-Bi2O3/g-C3N4 Heterojunction with Enhancing Photocatalytic Activity for Sulfamethoxypyridazine Degradation. Mater. Sci. Semicond. Process..

[44] Su C., Zhang N., Zhu X., Sun Z., Hu X. (2023). pH Adjustable MgAl@LDH-Coated MOFs-Derived Co2.25Mn0.75O4 for SMX Degradation in PMS Activated System. Chemosphere.

[45] Wang Y., Song Y., Li N., Liu W., Yan B., Yu Y., Liang L., Chen G., Hou L., Wang S. (2022). Tunable Active Sites on Biogas Digestate Derived Biochar for Sulfanilamide Degradation by Peroxymonosulfate Activation. J. Hazard. Mater..

[46] Chu Y., Xu M., Li X., Lu J., Yang Z., Lv R., Liu J., Lv L., Zhang W. (2024). Oxidation of Emerging Contaminants by S(IV) Activated Ferrate: Identification of Reactive Species. Water Res..

[47] Zhang Z., Li H., Liu J., Hu J., Wang S. (2025). Mechanistic Insights into the Role of N/O-Doped Biochar in Enhanced Phenolics Production during Biomass Pyrolysis. Green Energy Environ..

[48] Chen D., Wang H. (2019). HOMO–LUMO Gaps of Homogeneous Polycyclic Aromatic Hydrocarbon Clusters. J. Phys. Chem. C.

[49] Yu G., Wang Y., Cao H., Zhao H., Xie Y. (2020). Reactive Oxygen Species and Catalytic Active Sites in Heterogeneous Catalytic Ozonation for Water Purification. Environ. Sci. Technol..

[50] Wang Y., Duan X., Xie Y., Sun H., Wang S. (2020). Nanocarbon-Based Catalytic Ozonation for Aqueous Oxidation: Engineering Defects for Active Sites and Tunable Reaction Pathways. ACS Catal..

[51] Yin R., Guo W., Wang H., Du J., Wu Q., Chang J.-S., Ren N. (2019). Singlet Oxygen-Dominated Peroxydisulfate Activation by Sludge-Derived Biochar for Sulfamethoxazole Degradation through a Nonradical Oxidation Pathway: Performance and Mechanism. Chem. Eng. J..

[52] Zhou L., Yan Y., Mao H., Zhou S., Hui J., Li H., Li M., Zhao Y., Zhang Q., Xia S. (2023). Development of Attapulgite Based Catalytic Membrane for Activation of Peroxymonosulfate: A Singlet Oxygen-Dominated Catalytic Oxidation Process for Sulfamethoxazole Degradation. Sep. Purif. Technol..

[53] Hu J., Li X., Liu F., Fu W., Lin L., Li B. (2022). Comparison of Chemical and Biological Degradation of Sulfonamides: Solving the Mystery of Sulfonamide Transformation. J. Hazard. Mater..

