# Supplementary material for: In Situ Electrochemically Generating High-Valent Iron Species Activated by Nitrogen-Doped Biochar for Efficient Degradation of Antibiotics
Source: Antibiotics (Basel). 2026 Mar 1;15(3):254. doi: 10.3390/antibiotics15030254 (PMC13023536; doi:10.3390/antibiotics15030254)
Supplement: Supplementary file 1 [file antibiotics-15-00254-s001.zip › antibiotics-4148778-supplementary.pdf]

# Nitrogen-doped biochar activated in-situ electrochemically generating high-valent iron species for the efficient degradation of sulfamethoxazole

**TEXT S1**

Sulfamethoxazole (SMX,>99 %, Aladdin, Shanghai Aladdin Biochemical Technology Co., Ltd., Shanghai, China), methyl phenyl sulfoxide(PMSO, >98 %, Aladdin, Shanghai Aladdin Biochemical Technology Co., Ltd., Shanghai, China), methyl phenyl sulfone($PMSO_2$, >98 %, Aladdin, Shanghai Aladdin Biochemical Technology Co., Ltd., Shanghai, China)，sulfadiazine（SDZ, >99 %, Aladdin, Shanghai Aladdin Biochemical Technology Co., Ltd., Shanghai, China），sulfadoxine（SDX, >99 %, Aladdin, Shanghai Aladdin Biochemical Technology Co., Ltd., Shanghai, China），sulfamethoxypyridazine（SMP, >99 %, Aladdin, Shanghai Aladdin Biochemical Technology Co., Ltd., Shanghai, China）、sulfathiazole（STZ, >99 %, Aladdin, Shanghai Aladdin Biochemical Technology Co., Ltd., Shanghai, China）,Urea(>99 %, Aladdin, Shanghai Aladdin Biochemical Technology Co., Ltd., Shanghai, China), iron sulfate(>99 %, Aladdin, Shanghai Aladdin Biochemical Technology Co., Ltd., Shanghai, China), sodium tetraborate(>99 %, Aladdin, Shanghai Aladdin Biochemical Technology Co., Ltd., Shanghai, China), sodium thiosulfate pentahydrate(>99 %, Aladdin, Shanghai Aladdin Biochemical Technology Co., Ltd., Shanghai, China), boric acid(>99 %, Aladdin, , Shanghai Aladdin Biochemical Technology Co., Ltd., Shanghai, China)，tert-butanol (TBA, >99.8 %, Aladdin, Shanghai Aladdin Biochemical Technology Co., Ltd., Shanghai, China), furfuryl alcohol (FFA, >99.8 %, Aladdin, Shanghai Aladdin Biochemical Technology Co., Ltd., Shanghai, China), humic acid (HA, >99 %, Aladdin, Shanghai Aladdin Biochemical Technology Co., Ltd., Shanghai, China)、 benzoquinone (BQ, >99.8 %, Aladdin, Shanghai Aladdin Biochemical Technology Co., Ltd., Shanghai, China)、5,5-dimethyl-1-pyran N-oxide (DMPO, >99.8 %, Aladdin, Shanghai Aladdin Biochemical Technology Co., Ltd., Shanghai, China) and 2,2,6,6-tetramethyl-4-piperidinol (TEMP, >99.8 %, Aladdin, Shanghai Aladdin Biochemical Technology Co.,

Ltd., Shanghai, China)

**TEXT S2**

Residual SMX concentrations was obtained to determine the concentration of the remaining SMX using HPLC (Agilent 1200 Series, Agilent Technologies, Inc., USA) at a flow rate of 1.0 mL/min at a wavelength of 270 nm. The mobile phase was acetonitrile and 0.1 % formic acid with a ratio of 20:80 (V/V).

**Test S3**

PMSO and $PMSO_2$ were analyzed using HPLC (Agilent 1200 Series, Agilent Technologies, Inc., USA) at a flow rate of 1.0 mL/min at a wavelength of 230 nm and 215 nm, respectively. The mobile phase was acetonitrile and 0.1 % formic acid with a ratio of 20:80 (V/V).

**Test S4**

The concentration of SDZ、SMP、STZ and SDX were determined by HPLC (Agilent 1200 Series, Agilent Technologies, Inc., USA) at a flow rate of 1.0 mL/min. The isocratic mobile phases were water containing 0.1% formic acid and acetonitrile. The VWD detector was set at 270 nm for SMP, with a 60:40 ratio of formic acid water to acetonitrile. SDZ was detected at 254 nm with a 70:30 ratio, while SDX employed a 60: 40 ratio at 254 nm. STZ were quantified at 270 nm with a 65: 35 ratio.

Test S5（LC-MS）

Instrument Conditions

Liquid Chromatography: Agilent 1290 ULC

Mass Spectrometry: Agilent QTOF 6550

Mobile Phase A: 0.1% formic acid aqueous solution; Mobile Phase B: acetonitrile solution;

| Time/min | Phase A/% | Phase B/% |
|---|---|---|
| 0 | 95 | 5 |

| | | |
|---|---|---|
| 0.5 | 95 | 5 |
| 1 | 84 | 16 |
| 5 | 84 | 16 |
| 9 | 5 | 95 |
| 19 | 5 | 95 |
| 20 | 95 | 5 |
| 22 | 95 | 5 |

Flow rate: 0.3 ml/min

Injection volume: 5 μL

Column: Waters BEH C18 2.1 × 100 mm, 1.7 μm

MS scan range: Q1 100–600 m/z

Sheath gas temp: 350°C

Sheath gas flow: 12 L/min

ESI+ mode: Voltage 4000 V

**Test S6**

200 uL of the samples were mixed with 20 uL of spin-trapping agent (DMPO or TEMP) after 10 minutes of reaction and then analyzed using a spectrometer (JEOL FA200, Japan). EPR measurements were conducted using a radiation of 9.147 GHz (X band) with a modulation frequency of 100 kHz, modulation width of 0.1 mT, sweep width of 20 mT, center field of 326.0 mT, scan time of 60 s, time constant of 0.03 s, and microwave power of 5 mW.

**Table S1. Summary of degradation products in the EC/Fe(III)/NBC system.**

| transformation products (m/z) | Proposed formula | Proposed structure |
|---|---|---|
| 157 | $C_6H_8NO_2S^+$ | 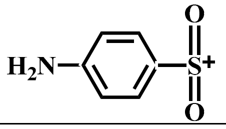 |
| 173 | $C_6H_7NO_3S$ | 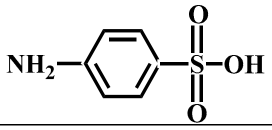 |
| 180 | $C_4H_8N_2O_4S$ | 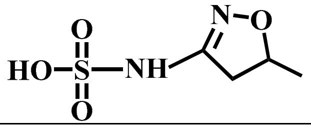 |
| 201 | $C_6H_6N_2O_4S$ | 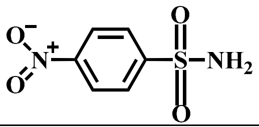 |
| 218 | $C_8H_{12}N_2O_3S$ | 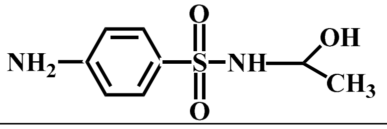 |
| 258 | $C_{10}H_{15}N_3O_3S$ | 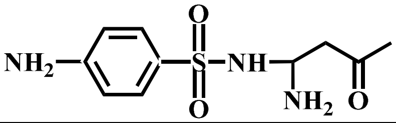 |
| 262 | $C_9H_{15}N_3O_4S$ | 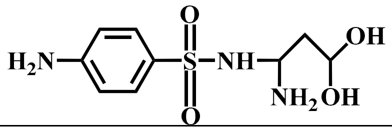 |
| 268 | $C_{10}H_{11}N_3O_4S$ | 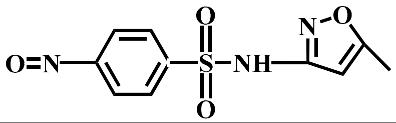 |
| 270 | $C_{10}H_{13}N_3O_4S^+$ | 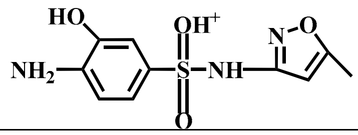 |
| 284 | $C_{10}H_{10}N_3O_5S$ | 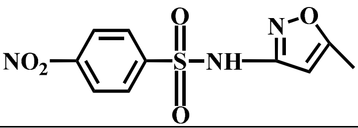 |
| 302 | $C_{10}H_{11}N_3O_6S$ | 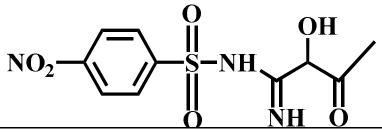 |

**Table S2 The toxicity levels of chemicals based on the Globally Harmonized System of Classification and Labelling of Chemicals**

| Toxicity range (mg/L) | Logarithmic-transformed toxicity range | Class |
|---|---|---|
| $k \leq 1$ | $\lg k \leq 0$ | Very toxic |
| $1 < k \leq 10$ | $0 < \lg k \leq 1$ | Toxic |
| $10 < k \leq 100$ | $1 < \lg k \leq 2$ | Harmful |
| $k > 100$ | $\lg k > 2$ | Not harmful |

*k*: Fish ($LC_{50}$), daphnid ($LC_{50}$), green algae ($EC_{50}$), or chronic toxicity

**Table S3 The toxicity values of SMX and degradation products (EC/Fe(III)/NBC)**

| Chemicals | Acute toxicity (mg/L) | | | Chronic toxicity (mg/L) | | |
|---|---|---|---|---|---|---|
| | Fish （$LC_{50}$） | Daphnid ($LC_{50}$) | Green Algae ($EC_{50}$) | Fish | Daphnid | Green Algae |
| SMX | 410.762 | 1.872 | 6.615 | 2.337 | 0.086 | 10.402 |
| 268 | 265.138 | 408.436 | 7.125 | 232.469 | 14.16 | 4.921 |
| 284 | 11514.412 | 50081.801 | 190.308 | 3.353 | 392.398 | 50.817 |
| 302 | $2.61e^{7}$ | $9.71e^{8}$ | $1.58e^{5}$ | 477.856 | $3.52e^{5}$ | 5926.242 |
| 201 | 44923.871 | $3.14e^{5}$ | 597.450 | 7.089 | 1246.532 | 102.765 |
| 157 | 52102.297 | 22631.148 | 5568.497 | 3712.922 | 1047.127 | 803.034 |
| 173 | $1.72e^{5}$ | 13.549 | 184.4558 | 1099.879 | 3.354 | 226.189 |
| 270 | 1539.254 | 190.522 | 1063.861 | 115.998 | 36.362 | 512.583 |
| 218 | 11145.557 | 51857.852 | 178.488 | 2.975 | 368.907 | 44.808 |
| 180 | 89331.828 | 5821.828 | 15861.355 | 32757.354 | 265.879 | 3407.338 |
| 258 | $1.84e^{5}$ | $1.79e^{6}$ | 2089.140 | 19.014 | 4432.214 | 267.301 |

**Figure. S1**

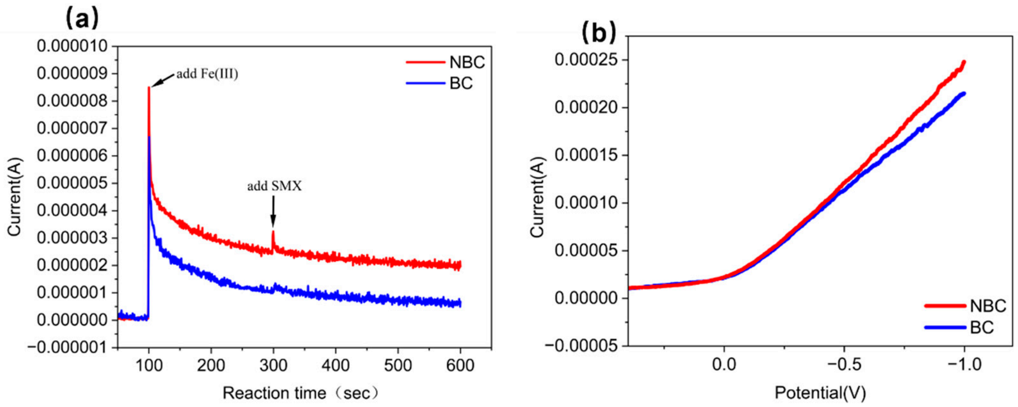


Figure S1. i-t (a) of BC and NBC(a)； LSV curves of BC and NBC(b)

**Figure. S2**

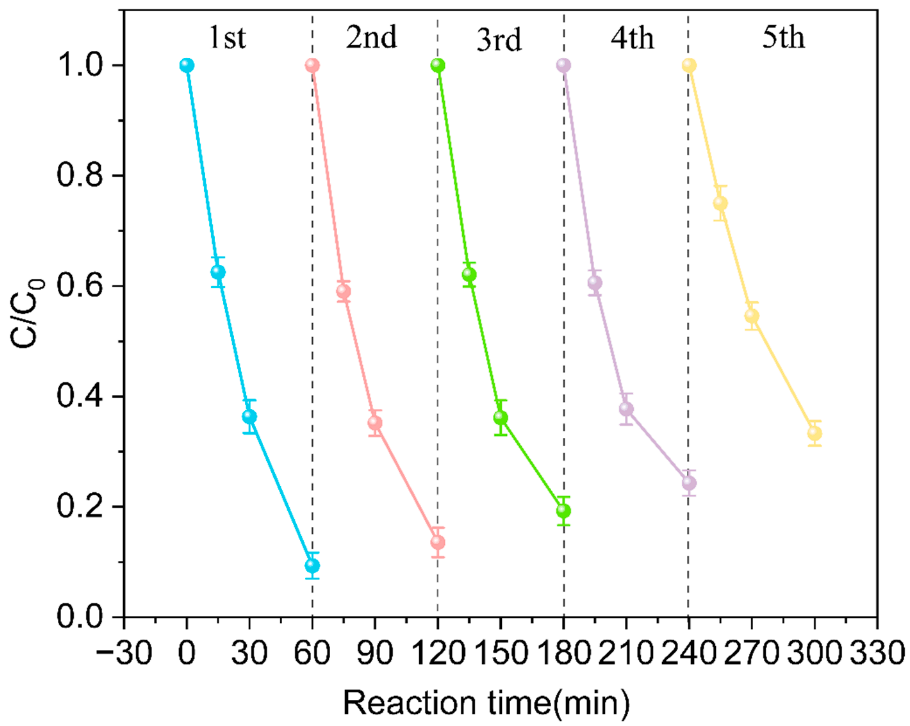


Figure S2. Cycle experiment of NBC ($C_0$(SMX)=0.04mM, C0($Fe^{3+}$) =25uM, E0=15V, m(NBC)=30mg, V=0.05 L, t=0–60min)

**Figure. S3**

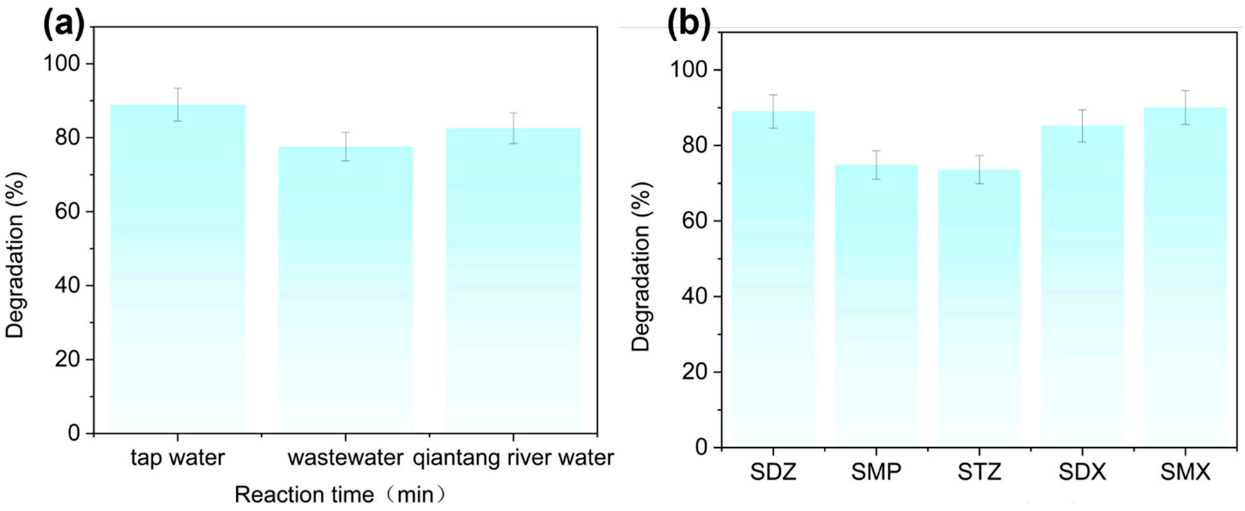


Figure S3. Removal of SMX in real water(a); The degradation performance of SAs(b)

**Figure. S4**

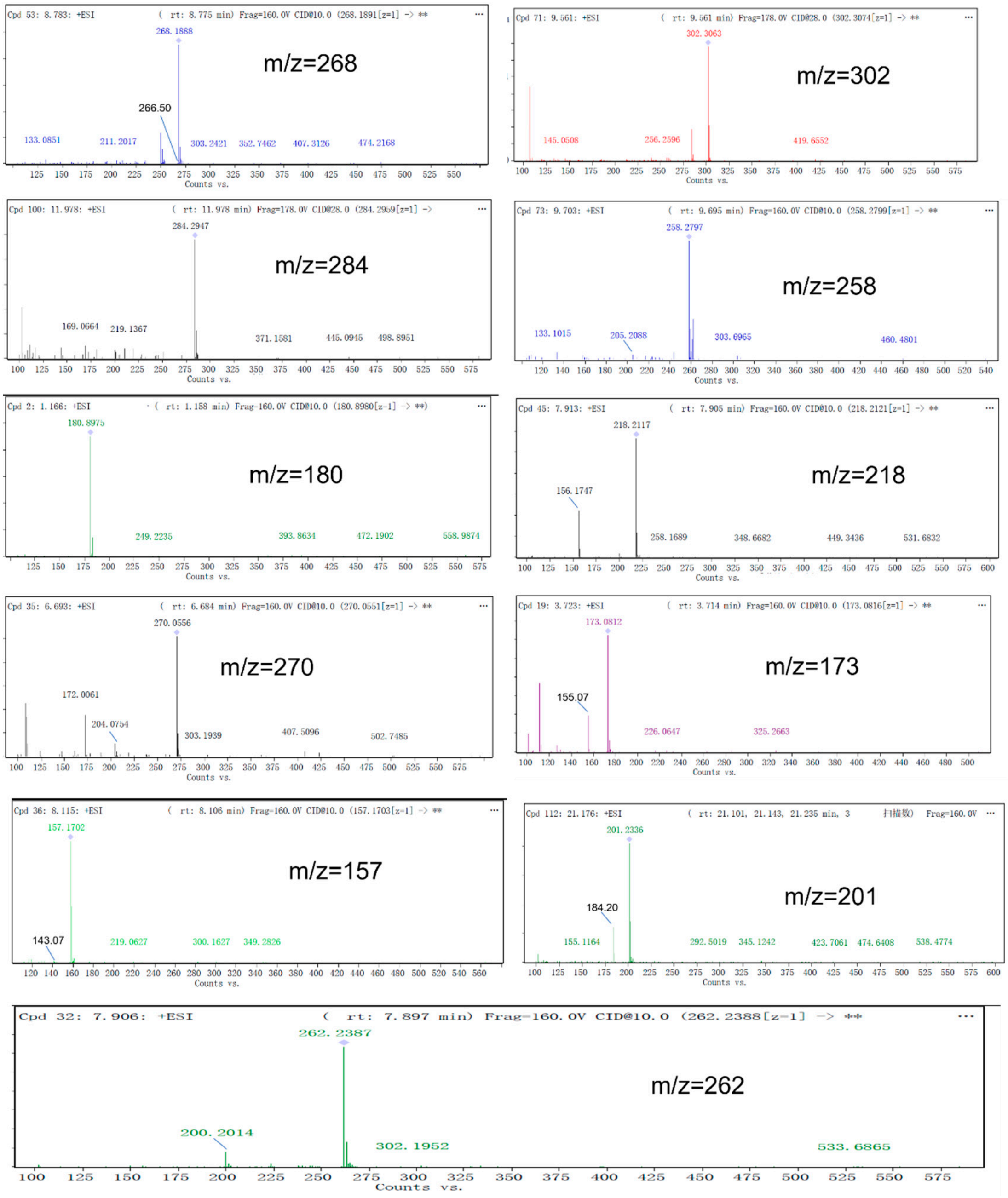


Fig.S4. Mass spectra of degradation products
